# The Potential of the Combination of CRISPR/Cas9 and Pluripotent Stem Cells to Provide Human Organs from Chimaeric Pigs

**DOI:** 10.3390/ijms16036545

**Published:** 2015-03-23

**Authors:** Wanyou Feng, Yifan Dai, Lisha Mou, David K. C. Cooper, Deshun Shi, Zhiming Cai

**Affiliations:** 1 Shenzhen Key Laboratory of Xenotransplantaton, State and Local Joint Cancer Genome Clinical Application of Key Technology Laboratory, Shenzhen Second People’s Hospital, First Affiliated Hospital of Shenzhen University, Shenzhen 518039, China; E-Mail: fwanyou@gmail.com; 2 Jiangsu Key Laboratory of Xenotransplantation, Nanjing Medical University, Nanjing 210029, China; E-Mail: daiyifan@njmu.edu.cn; 3 Thomas E. Starzl Transplantation Institute, University of Pittsburgh Medical Center, Pittsburgh, PA 15261, USA; E-Mail: coopdk@upmc.edu; 4 State Key Laboratory of Conservation & Utilization of Subtropical Agro-Bioresources, Guangxi University, Nanning 530005, China; E-Mail: ardsshi@gxu.edu.cn

**Keywords:** CRISPR/Cas9, genome editing, iPSC, pigs, chimaeric, xenotransplantation

## Abstract

Clinical organ allotransplantation is limited by the availability of deceased human donors. However, the transplantation of human organs produced in other species would provide an unlimited number of organs. The pig has been identified as the most suitable source of organs for humans as organs of any size would be available. Genome editing by RNA-guided endonucleases, also known as clustered regularly interspaced short palindromic repeat (CRISPR/Cas9), in combination with induced pluripotent stem cells (iPSC), may have the potential to enable the creation of human organs from genetically-modified chimaeric pigs. These could potentially provide an unlimited supply of organs that would not be rejected by the recipient’s immune system. However, substantial research is needed to prove that this approach will work. Genetic modification of chimaeric pigs could also provide useful models for developing therapies for various human diseases, especially in relation to drug development.

## 1. Introduction

Many patients with severe organ failure would benefit from organ transplantation, which would result in an improved quality of life and prolong their survival. However, the availability of suitable allografts limits access to organ transplantation and results in substantial waiting lists with a significant mortality during the waiting period. Recently-introduced technologies, especially interspecies chimaeras generated with pluripotent stem cells (PSC) or induced PSC (iPSC), suggest that it will soon be possible to produce human organs in genetically-modified chimaeric pigs [1,2,3]. iPSC derived from the patient who needs an organ could be injected into a genetically-modified pig embryo, enabling a human organ to develop which can subsequently be transplanted into the patient [4,5]. However, researchers face numerous difficulties in making human organs with this approach because the evolutionary distance between pig and human could prevent the successful development of interspecies blastocyst chimaeras. Other potential problems include the ethical issues of human-pig chimaeras and the potential for the generation of hybrid human-pig viruses. With the recent development of CRISPR/Cas9 technology, which significantly increases gene-editing efficiency, it is possible to test the approach of producing human organs through a combination of CRISPR/Cas9 and iPSC technologies.

## 2. Designer Endonucleases as a Tool for Precise and Efficient Gene Editing

**Figure 1:**
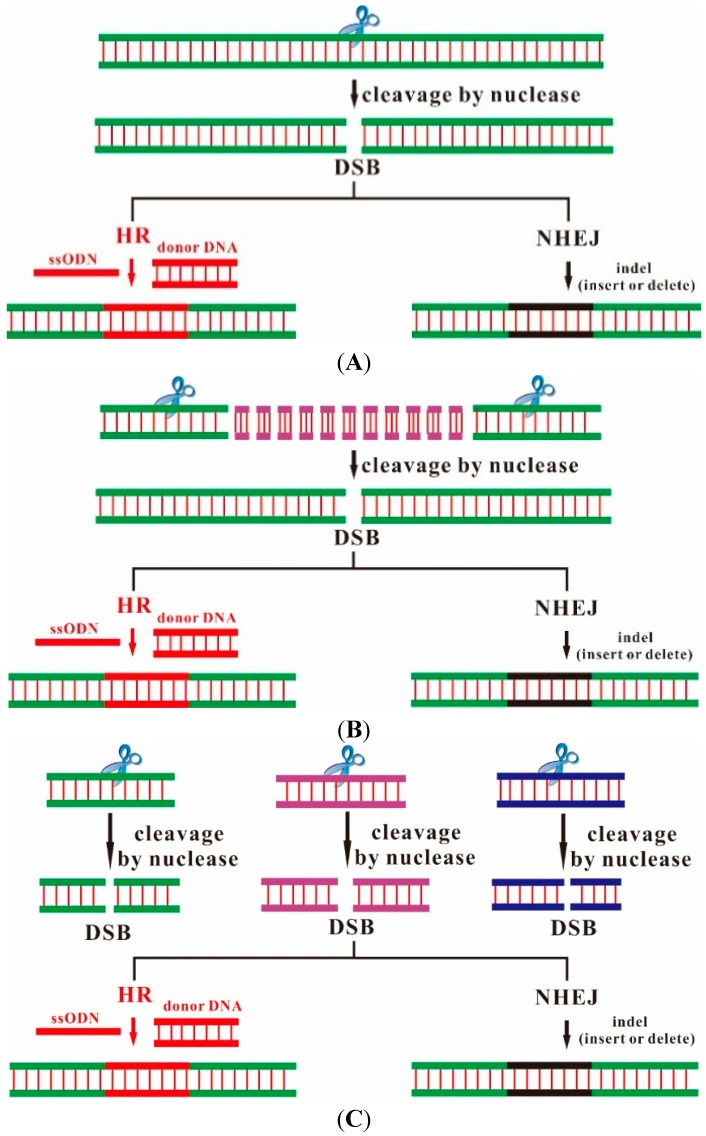
Outcome of genome editing used nucleases. Nuclease-induced DNA double-strand breaks (DSBs) can lead to sequence indels (insertion or deletion; black) through non-homologous end-joining (NHEJ) or nucleotide correction (red) through homology-directed repair (HR) in the presence of a donor DNA or a single-strand oligodeoxynucleotide (ssODN). (**A**) single gene editing; (**B**) long sequence deletion; and (**C**) multiple gene editing.

**Figure 2:**
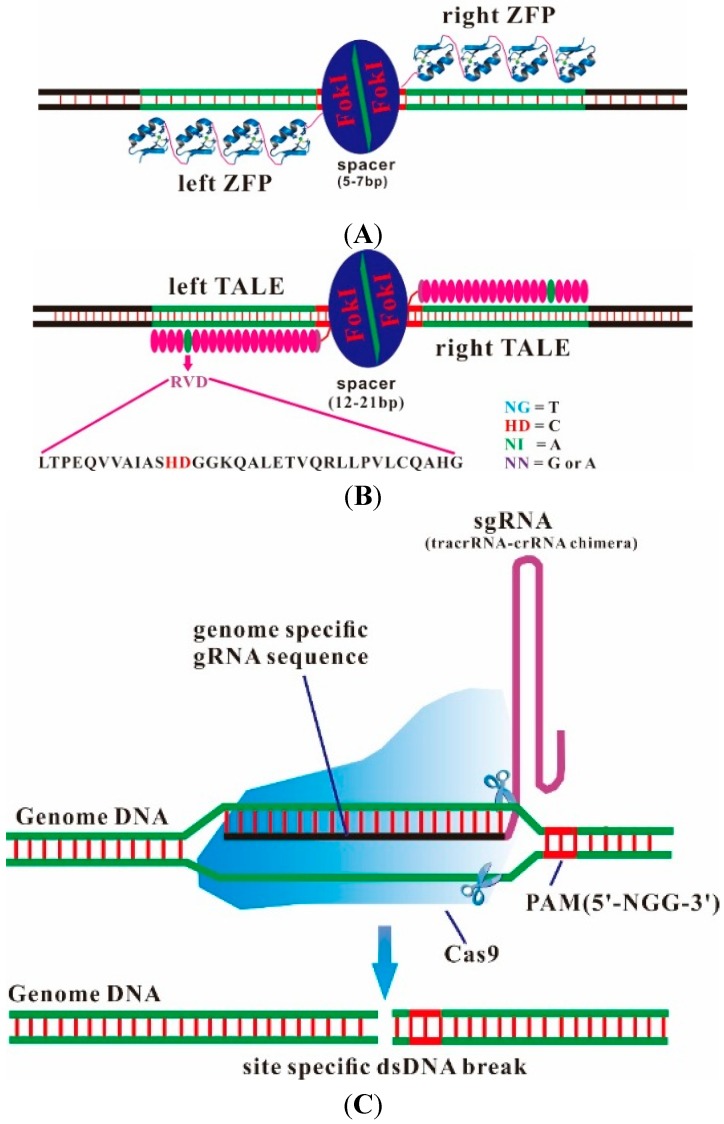
Schematic representation of (**A**) ZFN, (**B**) TALEN, and (**C**) CRISPR/Cas9. (**A**) Each ZFN is composed of different zinc-finger proteins (ZFP) at the amino terminus and of the FokI nuclease domain at the carboxyl terminus. Each ZFP recognizes three base pairs; (**B**) Each transcription activator-like effector nuclease (TALEN) is composed of a transcription activator-like effector (TALE) at the amino terminus and the FokI nuclease domain at the carboxyl terminus. Each TALE repeat is comprised of 33–35 amino acids and recognizes a single base pair through the amino acids at positions 12 and 13, which is called the repeat variable diresidue (RVD, shown in red); and (**C**) CRISPR/Cas9 is composed of Cas9 protein and a single-chain guide RNA (sgRNA). The guide sequence in the crRNA (shown in black) is complementary to a 20-bp target DNA sequence known as a protospacer, which is next to the 5'-NGG-3'.

A series of studies demonstrated that designer endonucleases, such as zinc finger nucleases (ZFNs) and transcription activator-like effector nucleases (TALENs), enable genetic modifications to be made by inducing DNA double-strand breaks that stimulate error-prone nonhomologous end joining or homology-directed repair at specific genomic locations [6] (Figure 1 and Figure 2A,B). ZFNs and TALENs recognize specific DNA sequences by protein-DNA interactions and induce DNA site-specific lesions through the dimeric nuclease domain of FokI. Each of these platforms, however, has unique limitations (Table 1). Although multiple strategies have been developed to overcome many limitations, fusing of functional ZFNs and TALENs still requires a time-consuming screening process [7,8,9,10,11,12,13,14].

The recent development of the CRISPR/Cas9 genome editing system, employing components of the bacterial adaptive immune response pathway, does not require custom protein synthesis, and instead uses a unique guide RNA along with a single endonuclease protein (Cas9) [15] (Table 1 and Figure 2C).

**Table 1:** Comparison of intrinsic technical performance for ZFN, TALEN and CRISPR/Cas9.

Factors	ZFN	TALEN	CRISPR/Cas9
Nuclease construction	significant	significant	simple
*In vitro* testing	significant	significant	simple
Target-efficient	limiting factor	average	good
Off-target-efficient	high	low	low
Target site choose	limited	limited	unlimited
Multiple gene mutations	limited	limited	unlimited
Designed component	protein	protein	RNA
Essential components	zinc finger proteins + FokI fusion protein	TALE and FokI fusion protein	guid RNA + Cas9 protein
Time consumption	long (7–15 days)	long (5–7 days)	short (1–3 days)
Cost	high	high	low

## 3. From Bacterial CRISPR Immune Systems to Engineered RNA-Guided Endonucleases (CRISPR/Cas9)

The CRISPR story began in 1987, when Ishino and coworkers discovered an unusual structure of repetitive DNA downstream from the *Escherichia coli* iap gene consisting of invariant direct repeats and variable spacing sequences; these invariant direct repeats were interspaced by five intervening variable spacing sequences [16]. Because of this feature, they received the name CRISPR (clustered regulatory interspaced short palindromic repeats). Furthermore, these CRISPR cassettes are located in close proximity to the CRISPR associated genes (Cas), the protein products of which have helicase and nuclease activity. Over 20 years, the basic function and mechanisms of CRISPR/Cas systems in bacteria have become clear. It has been proposed that CRISPR/Cas is an adaptive defense system that might use antisense RNAs as memory signatures of previous bacteriophage infection by exploiting Watson-Crick base pairing between nucleic acids. During the adaptation stage, resistance is acquired by integration of a new spacer sequence in a CRISPR array. During the expression stage, CRISPR arrays are then transcribed and processed into small RNAs (crRNAs) and Cas proteins. In the late interference stage, the crRNA guide Cas9 proteins to cleave complementary nucleic acids [17,18].

A key advance was the dual tracrRNA:crRNA in 2012, which was engineered as a single-guide RNA (suit guide RNA, sgRNA) that retained initial functionality. The 20-nucleotide sequence at the 5' end of the sgRNA determines the DNA target site by Watson-Crick base pairing, and the double-stranded structure at the 3' side of the guide sequence binds Cas9 to cleave any DNA sequence of interest, as long as it is adjacent to a protospacer-adjacent motif (PAM) (Figure 2C) [19]. In contrast to ZFNs and TALENs, which require substantial protein engineering and a time-consuming screening process for each DNA sequence of interest, the CRISPR/Cas9 system requires only a change in a 20-nucleotide sequence at the 5' end of the sgRNA [20,21,22]. The CRISPR/Cas9 technology has been rapidly and widely adopted to target genome editing of a vast array of cells and animals [23,24].

## 4. Rapid and Efficient Generation of Genetically-Modified Animals

CRISPR/Cas9-mediated genome editing has enabled accelerated generation of genetically-modified animals. In 2012, Jinek *et al.* demonstrated that dual tracrRNA:crRNA directed the CRISPR/Cas9 to introduce double-stranded breaks in the target DNA *in vitro* [19]. Three studies in January 2013 showed that CRISPR/Cas9 represented an efficient tool to edit the genomes of human cells with humanized versions of *Streptococcus pyogenes* Cas9 [20,21,22]. CRISPR/Cas9-mediated editing has been applied to various cells and animals [25,26,27,28,29,30,31,32,33]. For rapid and efficient generation of genetically-modified animals, Cas9 can be easily introduced into the target cells using transient transfection of plasmids which carry Cas9 and the appropriately designed sgRNA, followed by somatic cell nuclear transfer (SCNT) [32].

An alternative method involves Cas9 and sgRNA transcribed into mRNA *in vitro* and directly injected into fertilized zygotes to achieve heritable gene modification at one or multiple alleles (Figure1A,C) [30,34,35]. In order to simply and quickly develop genome editing in mouse models, four recent studies described a more convenient method of model generation using the CRISPR/Cas9 system *in vivo* in wild-type mice [36,37,38,39]. In 2014, Hai and Whitworth and their respective colleagues showed that zygote injection of the Cas9 and sgRNA mRNA efficiently generated genome-modified pigs in one step [29,34]. Rapid and efficient CRISPR/Cas9-mediated genome editing in pigs has opened up unlimited possibilities of genetic engineering in large animals for applications in regenerative medicine.

## 5. Making Human Organs from Human iPSCs and Genetically-Engineered Chimaeric Pigs

Four main methods of providing functional organs for humans have been reported in recent years: (i) creation of organs *in vitro* in the laboratory (“lab-dish” organs) from PSC or iPSC [40,41,42,43,44]; (ii) the construction of bionic organs *in vitro* [45,46,47]; (iii) *ex vivo* decellularization and recellularization of human or pig organs through regenerative techniques [48,49,50,51,52]; and (iv) genetic engineering of pigs to render their organs resistant to the human immune response (xenotransplantation) [53,54,55]. The *in vitro* generation of organs derived from PSC and iPSC or the construction of bionic organs is complex. Blastocyst complementation, first reported by Chen *et al.*, can provide the organ with a “developmental niche” *in vivo*, generating an almost entirely iPSC-derived organ [56]. They showed that deficiency of T and B lymphocyte lineages in Rag2-deficient mice was complemented by injecting normal mouse embryonic stem cells into mutant Rag2 mouse-derived blastocysts.

To examine the potential for xenogeneic approaches in blastocyst complementation, the groups of Nakauchi and Okabe demonstrated that interspecies chimaeras can be used for the generation of an entire organ from PSCs or iPSC [57,58]. In these studies, the researchers injected rat iPSC into a mutant mouse, which would normally be born lacking a pancreas or thymus, but the injection of wild-type rat iPSCs allowed the development of rat organs in these chimaeras. In theory, interspecies hybrids can be used to generate any tissue or organ type, regardless of its complexity. In 2013, Nakauchi and colleagues successfully generated a pig pancreas by blastocyst complementation, indicating that this approach is successful in a large animal [1].

**Figure 3:**
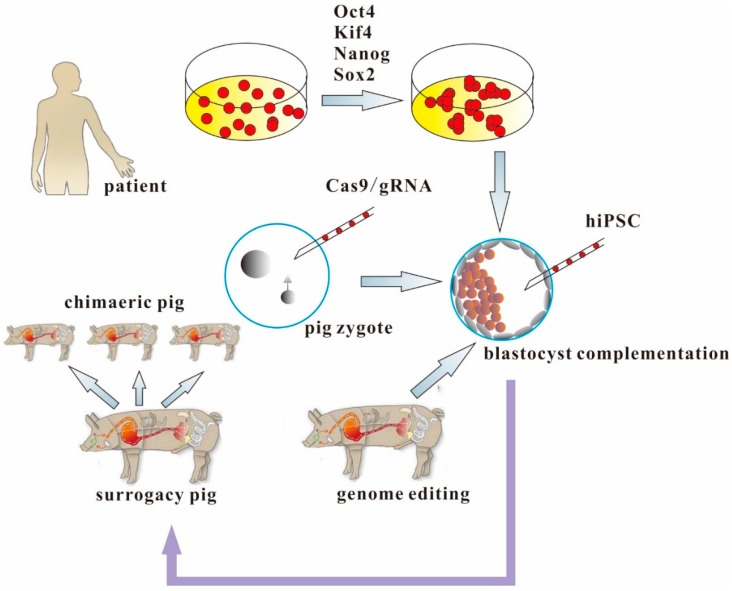
Combination of CRISPR/Cas9 and pluripotent stem cells to provide human organs from chimaeric pigs. Generation of human organs by producing multigene mutations of essential regulators of vascular and lymphatic tissues in the desired organ via rapid and efficient CRISPR/Cas9-mediated genome editing in concert with blastocyst complementation.

The feasibility of blastocyst complementation using cloned porcine embryos allows experimentation toward the *in vivo* generation of functional organs from xenogeneic PSCs in large animals. Following Nakauchi’s study, Kim reported that Rag2-mutant pigs (produced through TALENs, with a T-B-NK^+^ SCID phenotype) support proliferation and differentiation of human iPSCs and allogeneic porcine trophoblast stem cells [3]. However, it has yet to be proved that human organs can be generated in pigs by blastocyst complementation. In addition, to completely overcome the species barrier, with the associated risks of rejection and/or cross-species infection, one problem that is still to be overcome is the need to humanize the animal’s vascular system, which would still express pig antigens (Figure 3). This goal could be achieved by producing multi-gene mutations of essential regulators of vascular and lymphatic tissues in the desired organ via rapid and efficient CRISPR/Cas9-mediated genome editing in concert with blastocyst complementation.

However, two questions still remain in this field. First, the currently-available human iPSCs are so-called “primed” and do not develop chimeras when injected into blastocysts. It will be necessary to generate naïve human iPSCs to develop human/pig chimeras [59,60,61]. Second, safety and ethical issues remain in respect to developing human/pig chimeras. Human iPSC-derived chimeras would possibly carry human neural and germ cells, which evokes ethical controversy. However, with the advantage of CRISPR/Cas9, forced expression of specific genes can be used to guide human iPSC to target organs after blastocyst injection. This method has been successfully used to generate functional pancreas in pancreatogenesis-disabled Pdx1-knockout mice [5,62].

## 6. Conclusions

Organs from humanized chimaeric pigs that have undergone CRISPR/Cas9 system-mediated gene editing in concert with multigene blastocyst complementation have the potential to resolve the current problem of organ availability for purposes of transplantation. Furthermore, this combined technology could supply organs that will not trigger any immune response, and potentially save hundreds of thousands of lives each year. However, numerous challenges remain. Close collaboration between scientists and clinicians, and between academia and industry, will be required if the development of this technology is to succeed.

## Abbreviations

CRISPR: Clustered regularly interspaced short palindromic repeats
Cas: CRISPR-associated
PSC: pluripotent stem cells
TALENS: transcription activator-like effector nucleases
ZFNs: zinc-finger nucleases
